# A microbiological survey of handwashing sinks in the hospital built environment reveals differences in patient room and healthcare personnel sinks

**DOI:** 10.1038/s41598-020-65052-7

**Published:** 2020-05-19

**Authors:** Lauren C. Franco, Windy Tanner, Christine Ganim, Terri Davy, Jonathan Edwards, Rodney Donlan

**Affiliations:** 1 Centers for Disease Control and Prevention, Atlanta, GA USA; 2 University of Utah, Salt Lake City, UT USA; 3 Oak Ridge Institute for Science and Education, Oak Ridge, TN USA

**Keywords:** Pathogens, Antimicrobial resistance, Infection, Water microbiology, Biofilms

## Abstract

Handwashing sinks and their associated premise plumbing are an ideal environment for pathogen-harboring biofilms to grow and spread throughout facilities due to the connected system of wastewater plumbing. This study was designed to understand the distribution of pathogens and antibiotic resistant organisms (ARO) within and among handwashing sinks in healthcare settings, using culture-dependent methods to quantify *Pseudomonas aeruginosa*, opportunistic pathogens capable of growth on a cefotaxime-containing medium (OPP-C), and carbapenem-resistant Enterobacteriaceae (CRE). Isolates from each medium identified as *P. aeruginosa* or Enterobacteriaceae were tested for susceptibility to aztreonam, ceftazidime, and meropenem; Enterobacteriaceae were also tested against ertapenem and cefotaxime. Isolates exhibiting resistance or intermediate resistance were designated ARO. Pathogens were quantified at different locations within handwashing sinks and compared in quantity and distribution between healthcare personnel (HCP) and patient room (PR) sinks. ARO were compared between samples within a sink (biofilm vs planktonic samples) and between sink types (HCP vs. PR). The drain cover was identified as a reservoir within multiple sinks that was often colonized by pathogens despite daily sink cleaning. *P. aeruginosa* and OPP-C mean log_10_ CFU/cm^2^ counts were higher in p-trap and tail pipe biofilm samples from HCP compared to PR sinks (2.77  ± 2.39 vs. 1.23 ± 1.62 and 5.27 ± 1.10 vs. 4.74 ± 1.06) for *P. aeruginosa* and OPP-C, respectively. *P. aeruginosa* and OPP-C mean log_10_ CFU/ml counts were also higher (p < 0.05) in HCP compared to PR sinks p-trap water (2.21 ± 1.52 vs. 0.89 ± 1.44 and 3.87 ± 0.78 vs. 3.21 ± 1.11) for *P. aeruginosa* and OPP-C, respectively. However, a greater percentage of ARO were recovered from PR sinks compared to HCP sinks (p < 0.05) for Enterobacteriaceae (76.4 vs. 32.9%) and *P. aeruginosa* (25.6 vs. 0.3%). This study supports previous work citing that handwashing sinks are reservoirs for pathogens and ARO and identifies differences in pathogen and ARO quantities between HCP and PR sinks, despite the interconnected premise plumbing.

## Introduction

From 2014 to 2017, 21.6% of Centers for Disease Control and Prevention (CDC), Division of Healthcare Quality Promotion consultations involved water-related healthcare-associated infections (HAI). Among these investigations, 29.9%, 18.7%, and 10.4% were attributed to nontuberculous mycobacteria (NTM), *Pseudomonas* spp., and members of the Enterobacteriaceae family, respectively^1^. Plumbing fixtures, such as sinks, showers and toilets, with which patients, visitors, and healthcare personnel (HCP) interact are known reservoirs for pathogenic and opportunistic organisms^2–4^. Previous work has documented the role that these plumbing fixtures have played in outbreaks, many of which have involved gram-negative rods, such as *Pseudomonas* spp. and carbapenem-resistant or extended-spectrum β-lactamase producing (ESBL) Enterobacteriaceae^5–9^.

Handwashing sinks have been a target for infection control improvement in the healthcare environment because the nature of their design brings potentially high concentrations of healthcare pathogens such as the nontuberculous mycobacteria, *Pseudomonas aeruginosa*, and other Gram-negative bacteria into close proximity to patients, HCP, and fomites. In a study that analyzed the number of different activities performed at or near handwashing sinks in patient rooms, handwashing only accounted for 4% of sink activities. Other activities performed were associated with medical care, placing items near the sink, patient nutrition, or environmental cleaning^10^. The splash zone around a sink refers to the surfaces and objects nearby that may become contaminated by droplet mediated dispersion of pathogens following the impact of faucet water with a contaminated drain cover or sink basin^11^. Sink drain covers can become contaminated by patient or HCP input or by pathogen-containing biofilms that can grow from the p-trap to the drain cover, highlighting the importance that biofilm growth mode plays in the dissemination of organisms in premise plumbing systems^12^.

The luminal surface of piping provides an excellent environment for biofilm growth in many different scenarios, from oil pipelines to water distribution systems and wastewater plumbing^13–15^. The flow of liquid through the system provides shear force, possible delivery of carbon and energy sources, and a mechanism for detachment and distribution of organisms throughout the system, which in turn allows for the attachment, growth, and dissemination of pathogens throughout the wastewater plumbing system^12,16^. Additionally, biofilms have been shown to have enhanced rates of genetic exchange, posing a threat for increased transmission of mobile genetic elements containing antibiotic resistance genes between organisms in sink drains^17,18^.

In this study, different components of handwashing sinks, from the incoming tap water to the p-trap biofilm, were sampled in two hospitals and cultured to quantify target organisms of interest. Incoming tap water quality was monitored to determine if disinfectant residual or microbiological quality affected presence or quantity of pathogens in the sink drain samples. Quantities of pathogens on different surfaces within the sink were compared to identify specific areas of high bioburden. Sinks from patient rooms and HCP work stations were surveyed to assess the impact that patient use and/or proximity has on the quantities of pathogens present in the sinks. Antibiotic susceptibility of isolates was compared between sinks and sample types. Previous studies have examined the role sinks play in HAI, but this study details the contribution of individual sink and premise plumbing components and the role that HCP sinks play as a reservoir.

## Results

### Tap water quality

Tap water at each sink sampled was monitored for disinfectant residual, temperature, and quantity of heterotrophic bacteria (heterotrophic plate count, HPC). Both hospitals received free chlorine-treated drinking water from the same municipal source and water quality parameters are listed in Table 1. Free chlorine was detected in all tap water samples and ranged from 0.38–0.67 mg/L at Hospital 1 and 0.13–0.72 mg/L at Hospital 2. Hospital 1 used supplemental copper-silver ionization disinfection for the hot water system (target range 0.3–0.8 ppm Cu, 30–80 ppb Ag) and Hospital 2 used supplemental chlorine dioxide that ranged from 0.14–0.42 mg/L. HPC for the first catch samples ranged from 8 ×10^0^–1.56 × 10^3^ CFU/mL at Hospital 1 and 3 × 10^0^ – 1.45 × 10^4^ CFU/mL at Hospital 2. The presence or absence of aerators significantly impacted the first catch HPC at Hospital 1 where two of the sinks had aerators and two did not (45 ± 54 CFU/ml for sinks without aerators and 489 ± 520 CFU/ml for sinks with aerators, p-value = 0.033 from Paired Student’s T-test). Two-minute flush samples did not differ significantly between sinks with and without aerators (4.2 ± 6.2 CFU/ml for sinks without aerators and 2.7 ± 2.0 CFU/ml for sinks with aerators, p-value = 0.46 from Paired Student’s T-test). All sink faucets at Hospital 2 had aerators. Two-minute flush samples ranged from <6–21 CFU/ml at Hospital 1 and <6–3.5 × 10^3^ CFU/mL at Hospital 2, with two instances in which the HPC for the two-minute flush samples at Hospital 2 were above the EPA recommended 500 CFU/mL HPC guideline for drinking water sampled at a municipal distribution point^19^; the water quality at both hospitals was acceptable overall. Rapid growing nontuberculous mycobacteria were not present or below the limit of detection of 6 CFU/ml in first catch and two-minute flush samples. Temperature of the incoming water fluctuated from 11.5–14.4 °C at Hospital 1 and 14.7–22.5 °C at Hospital 2. The difference in temperature ranges between the two hospitals may be due to the time of year during which samples were collected.

**Table 1:** Tap water parameters pertaining to microbiological water quality. Values represent the four sinks sampled during the intensive sampling period from each hospital. Limit of detection was 6 CFU/ml.

	Hospital 1		Hospital 2	
	Mean	Range	Mean	Range
1st Catch HPC (CFU/ml)	267	8–1560	2371	3–14476
2-minute Flush (CFU/ml)	3	<6–21	182	<6–3514
Total Chlorine (mg/L)	0.59	0.44–0.73	0.61	0.22–0.78
Free Chlorine (mg/L)	0.52	0.38–0.67	0.52	0.13–0.72
Chlorine Dioxide (mg/L)	N/A	N/A	0.22	0.14–0.42
Temperature (°C)	12.5	11.5–14.4	18.3	14.7–22.5

## Distribution of ARO and pathogens within sinks

**Figure 1:**
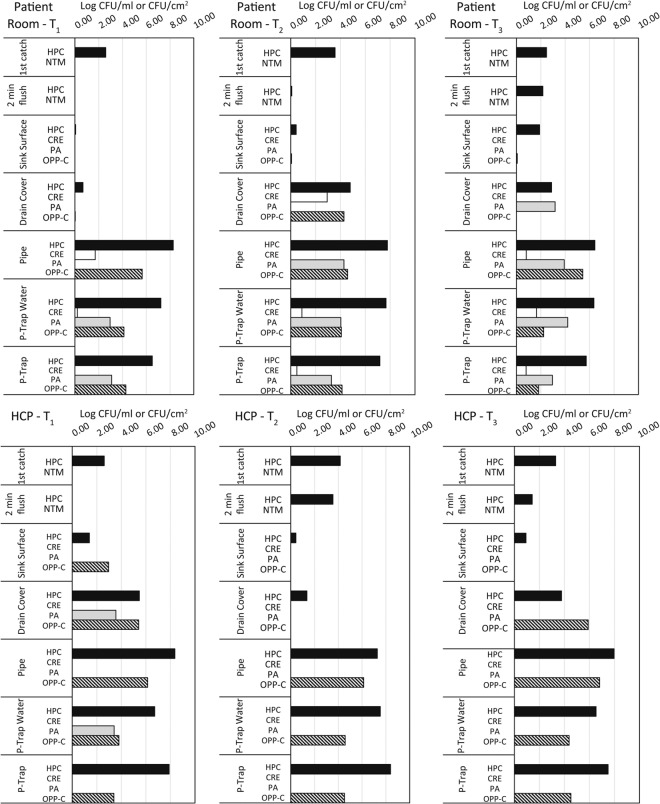
Distribution of target organisms in a patient room sink and HCP sink at Hospital 2. Patient room T_1_, T_2_, and T_3_ and HCP sink T_1_, T_2_, and T_3_ represent the same sinks sampled at week 0 (T_1_), week 3 (T_2_) and week 6 (T_3_). HPC – heterotrophic plate count, CRE – carbapenem-resistant Enterobacteriaceae, PA – *Pseudomonas aeruginosa*, OPP-C – opportunistic pathogens capable of growth on a cefotaxime-containing medium.

Figure 1 shows the distribution of target organisms in one patient room sink and one HCP sink over the six-week intensive sampling period at Hospital 2. In most instances, we observed high concentrations of target organisms in samples below the drain cover, which is expected, as surfaces above the drain cover (drain cover and sink surface) were cleaned daily. The drain cover varied in concentration level of colonization, and sometimes was heavily colonized compared to other sample time points (i.e. Patient room at T_1_ vs. T_2_). The typically high concentrations of target organisms in samples below the drain cover (tail pipe, p-trap biofilm, and p-trap water) suggests that this environment is ideal for biofilm formation and colonization by pathogens and ARO. Table S1 shows the isolate identifications and concentrations for all organisms that were isolated. *Stenotrophomonas maltophilia* was one of the most commonly isolated organisms and grew on many of the selective media plates. Although it is known to have many intrinsic antimicrobial resistance mechanisms, it was not one of the target organisms for this study and therefore was not further evaluated. *Sphingobacterium multivorum* and *S. spiritivorum* were frequently isolated from Chromagar KPC and mis-identified as members of Enterobacteriaceae based on colony morphology. When MALDI-ToF results revealed this misidentification, plate count numbers were adjusted so that counts reflect instances in which CRE were isolated from samples. Organisms that grew on MacConkey with cefotaxime agar (OPP-C) consisted of *Pseudomonas* spp (including *P. aeruginosa*), Enterobacteriaceae, *Achromobacter* spp., *Stenotrophomonas maltophilia*, *Acinetobacter* spp., and other organisms that comprised <1.5% of isolates recovered from this medium.

**Figure 2:**
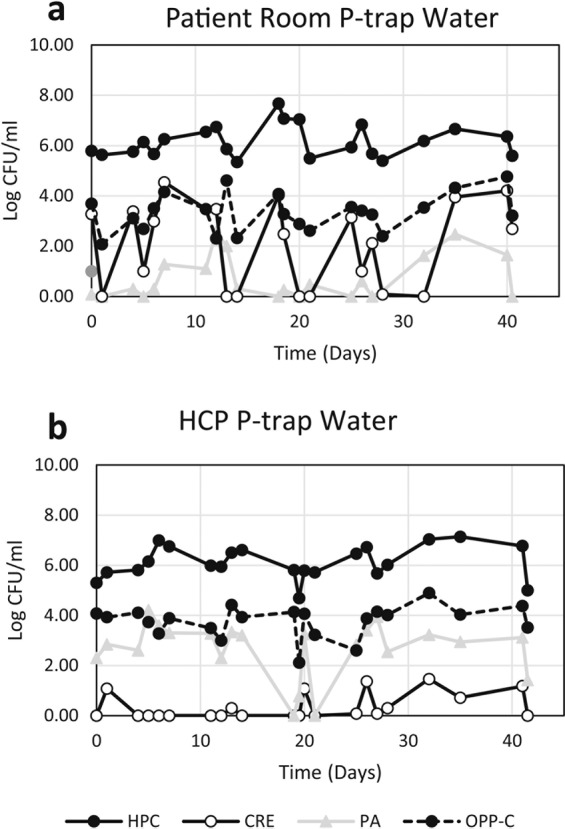
Concentration of target organisms from 4 times weekly p-trap water samples from one patient room sink (**a**) and one HCP sink (**b**) over the six-week sample period. HPC – heterotrophic plate count, CRE – carbapenem-resistant Enterobacteriaceae, PA – *Pseudomonas aeruginosa*, OPP-C – opportunistic pathogens capable of growth on a cefotaxime-containing medium.

**Figure 3:**
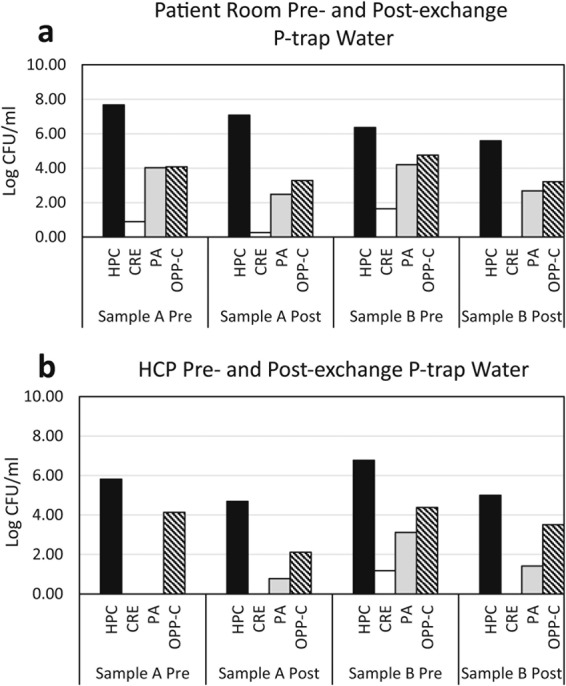
Concentration of target organisms in p-trap water pre- and post- p-trap exchange for sterilized p-trap in patient room (**a**) and HCP sinks (**b**). HPC – heterotrophic plate count, CRE – carbapenem-resistant Enterobacteriaceae, PA – *Pseudomonas aeruginosa*, OPP-C – opportunistic pathogens capable of growth on a cefotaxime-containing medium.

Concentrations of target organisms in the 4 times weekly p-trap water samples fluctuated from sample to sample within a sink (Fig. 2). The other sinks sampled displayed similar variation in concentration of target organisms. When p-trap water was sampled before and after the p-trap was exchanged with a sterile p-trap, the post-exchange p-trap water typically mirrored the pre-exchange water in composition of the target organisms (Fig. 3).

## Long-term p-trap colonization in Hospital 2

**Figure 4:**
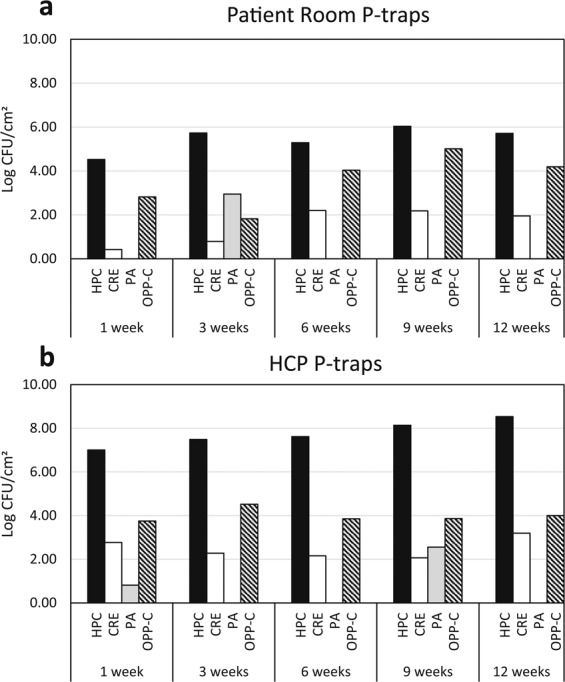
Concentration of target organisms from p-trap biofilms harvested from p-traps that had been in place at Hospital 2 in a patient room sink (**a**) and a HCP sink (**b**) for 1, 3, 6, 9, and 12 weeks. HPC – heterotrophic plate count, CRE – carbapenem-resistant Enterobacteriaceae, PA – *Pseudomonas aeruginosa*, OPP-C – opportunistic pathogens capable of growth on a cefotaxime-containing medium.

Sterilized p-traps were attached to the sinks and were left for 1, 3, 6, 9, and 12 week periods of time to determine how the concentration of target organisms changed over time. There was not a clear trend of increasing concentration of target organisms with increased incubation time and in some instances, the concentration of target organisms at one week was similar to the concentration at 12 weeks (Fig. 4b – CRE and OPP-C). In addition, some of

the target organisms were transient or below the limit of detection (LoD) in the biofilm, such as *P. aeruginosa* in Fig. 4b where it was present after 1 week and then below LoD until the 9-week sample.

## Comparing HCP and patient sinks

**Figure 5:**
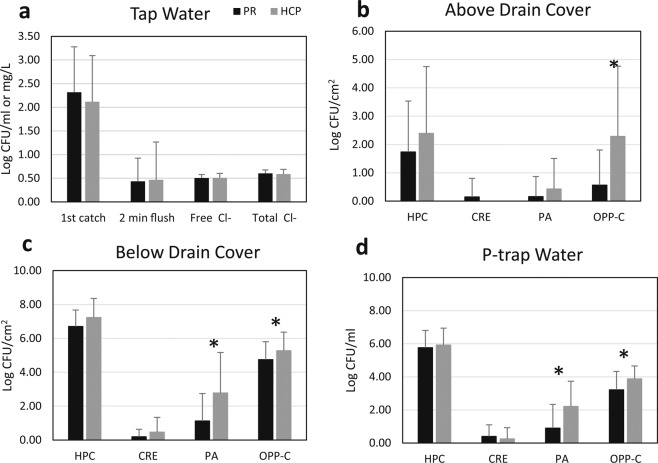
Target organism and free/total chlorine concentrations and in healthcare personnel (HCP) and patient room (PR) sinks for tap water (**a**), above drain cover (**b**), below drain cover (**c**), and p-trap water (**d**) samples. Asterisk (*) indicates p-value <0.05 from Wilcoxon signed-rank test. HPC – heterotrophic plate count, CRE – carbapenem-resistant Enterobacteriaceae, PA – *Pseudomonas aeruginosa*, OPP-C – opportunistic pathogens capable of growth on a cefotaxime-containing medium.

There were no significant differences between the incoming tap water quality in patient room vs. HCP sinks (p = 0.599 for first-catch HPC, p = 0.946 for 2-minute flush HPC, p = 0.903 for free chlorine, n = 32 samples) (Fig. 5a). Despite the similarity in incoming water quality, there were some notable trends in concentration of target organisms between HCP and patient room sinks. Samples were grouped into three categories: above drain cover (sink surface and drain cover samples, n = 38 samples), below drain cover (pipe and p-trap biofilm samples, n = 38 samples), and p-trap water (n = 128 samples). Above the drain cover samples had higher mean CFU/cm^2^ in HCP sinks compared to above drain cover samples from patient room sinks for all target organisms except CRE (Fig. 5b), but these differences were only statistically significant for OPP-C (p = 0.004). Below the drain cover, HCP sinks also had higher mean CFU/cm^2^ for all target organisms, with differences statistically significant for all target organisms except for CRE (HPC, p = 0.044; CRE, p = 0.195; PA, p = 0.048, OPP-C, p = 0.023) (Fig. 5c). P-trap water followed a similar trend, with HCP sinks having a higher mean CFU/cm^2^ for all target organisms, except for CRE (Fig. 5d). HCP sinks had significantly more *P. aeruginosa*, and OPP-C compared to patient room sinks in the p-trap water samples (HPC, p = 0.178; CRE, p = 0.139; PA, p = 0.001, OPP-C, p = 0.001).

## Antibiotic susceptibility of isolates from sink samples

**Figure 6:**
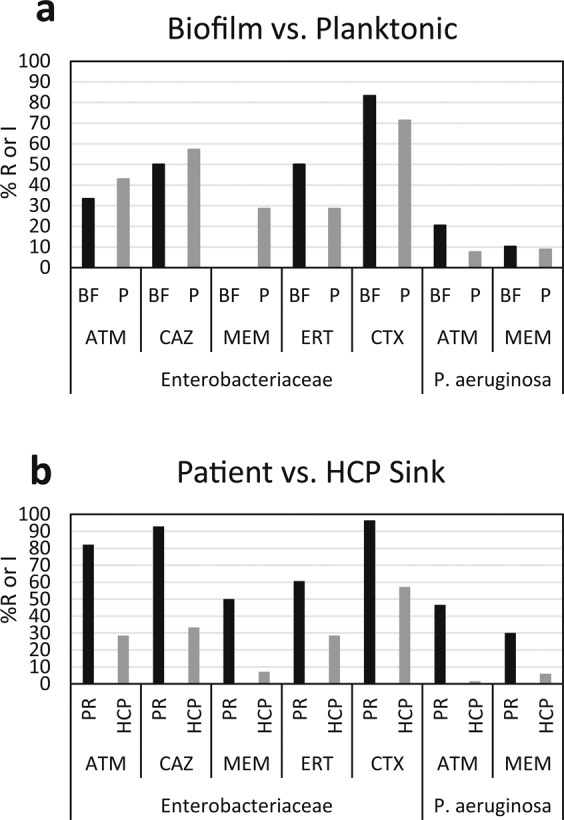
Percentage of Enterobacteriaceae and *P. aeruginosa* isolates that are resistant (R) or have intermediate (I) resistance to the antibiotics tested. Isolates originating from biofilm (BF, p-trap and tail pipe) were compared to those from planktonic (P, p-trap water) samples (**a**) and isolates originating from HCP sinks were compared to those from patient room sinks (**b**). ATM – aztreonam, CAZ – ceftazidime, MEM – meropenem, ERT – ertapenem, CTX – cefotaxime.

*P. aeruginosa* and Enterobacteriaceae (*Enterobacter* spp.*, Klebsiella* spp., *and Citrobacter* spp.) were the most frequently isolated pathogens (Table S1). Among these isolates (195 *P. aeruginosa* and 52 Enterobacteriaceae isolates), 28.5% of *P. aeruginosa* and 85.7% of Enterobacteriaceae were non-susceptible to one or more of the antibiotics tested. There were no significant differences in the percentage of ARO isolated from biofilm samples (from p-trap and tail pipe) compared to planktonic (p-trap water) samples (p = 0.915 for *P. aeruginosa* and p = 0.641 for Enterobacteriaceae) (Fig. 6a). There was a greater percentage of ARO recovered from patient room sinks compared to HCP sinks (p = 0.002 Enterobacteriaceae, p = 0.038 *P. aeruginosa*) for both *P. aeruginosa* and Enterobacteriaceae isolates (76.4 vs. 32.9% for Enterobacteriaceae, 25.6 vs 0.3% for *P. aeruginosa*) (Fig. 6b).

None of the *P. aeruginosa* isolates were resistant/intermediate to ceftazidime and they are not shown in the figure.

## Discussion

Sinks have been recognized as important reservoirs for pathogens in healthcare settings, but the distribution of pathogens within sinks and between HCP and patient room sinks has not been previously studied. In this study, individual samples from within the same sink varied in the level of target organism contamination. Because the sinks are cleaned regularly, the sink surfaces and drain covers were not as heavily contaminated as the pipe and p-trap surfaces. One limitation of this study is the time at which sinks were cleaned relative to when the sinks were sampled is unknown, but the variation in drain cover contamination between samplings suggests that the drain cover can become contaminated between cleanings and therefore may be an important locale in which to focus infection prevention practices, especially given the previous finding suggesting that bacteria must be present on the sink bowl surface or drain cover in order for dispersion to occur^11^. Additional variables such as sink usage and carbon and energy inputs were also unknown. Another limitation of this study is that it is unknown whether the source of drain cover contamination is from the biofilm in the plumbing below, or from patient, HCP, or visitor inputs during sink usage. Based on the findings of Kotay *et al*. demonstrating green fluorescent protein-labeled-*E. coli* growing from p-trap water to the drain cover in a span of seven days (eight-inch distance), contamination of the drain cover by the biofilm growing in the plumbing between daily cleanings is plausible^12^.

Heterotrophic plate counts from first catch samples at Hospital 1 varied significantly and this variation was associated with the presence or absence of faucet aerators. Faucet aerators have been previously documented as reservoirs for HAI pathogens^20,21^ and this finding further highlights the role that faucet aerators can play in promotion of biofilm growth. While there was a significant difference in HPC for first catch samples, no difference was observed in two-minute flush samples between faucets with and without aerators, indicating that the overall water quality was not compromised by the presence of aerators.

P-trap water samples taken 4 times per week also showed a large amount of variation in the concentration of target organisms present on a day-to-day basis. This variation may be due to sink usage including flushing, cleaning, or disposal of liquid waste materials (carbon or energy sources for microorganisms) down the sink drain^10^. Sampling of the p-trap water immediately before and after exchange of the in-place p-trap with a sterile p-trap gave insight into colonization dynamics within the premise plumbing. The data suggest that p-trap water is inoculated by the biofilm present on the drain cover, tail pipe, and p-trap or any other colonized surfaces that the water contacts as it flows from the faucet to the p-trap. The flow of water down the drain may be enough to dislodge large aggregates of biofilm and in some instances cause the post exchange p-trap water to have higher concentrations of an organism than the pre-exchange water. When ARO from biofilm and p-trap water were compared, there was no statistically significant difference between the two groups, further supporting the hypothesis that the microbiological composition of the p-trap water is largely related to the composition of the biofilms growing on the luminal pipe surfaces.

The long-term portion of this study aimed to look at the succession of the target organisms over time and if or when they reach a maximum carrying capacity or steady state. The high concentration of target organisms after one week compared to twelve weeks suggests that the new p-traps are colonized and the biofilm is established very quickly by the biofilm in the adjacent attached piping. Molecular methods may be better suited to detect how the community of the biofilm changes over time, but these culture-based results further support the argument against replacing plumbing fixtures as a way to eradicate pathogens from premise plumbing environments.

Another trend that emerged from this study was the difference in concentration of target ARO between HCP and patient room sinks. The higher concentration of the target organisms in HCP sinks compared to patient room sinks was unexpected and makes it apparent that the same safeguards and cleaning protocols should be practiced with HCP sinks as are practiced with sinks in patient rooms. The opposite trend emerged when the percentage of ARO was compared between HCP and patient room sinks. The higher percentage of ARO in patient room sinks compared to HCP sinks suggests that the source of the isolates may be different (i.e. patient) or that there is more selective pressure in patient room sinks due to antimicrobials or other waste material being disposed down the drain. A study that compared antibiotic resistance in patient isolates and environmental isolates obtained from the same hospital showed a similar trend in that a greater percentage of patient isolates were non-susceptible compared to the environmental isolates^4^. This raises the question of how patients may influence the microbial community of the hospital built environment. While it has been shown that patient room surfaces become increasingly similar to the microbiome of the patient occupying the room over the course of their stay, it has not been determined if this is true for patient room sinks as well^22^.

Findings from this study have identified areas of high bioburden within sinks and the associated premise plumbing that pose a risk for dispersion of pathogens to the surrounding environment. The proximity of high concentrations of target organisms in the tail pipe sample below the drain cover is one locale within sinks that could be the focus of a future infection control strategy. The dynamics of drain cover colonization, disinfection through daily cleaning, and recolonization from the tail pipe biofilm is a process that should be further explored to better understand and prevent dispersion of pathogens from sink drains to the patient environment. Replacement or harsh cleaning of sink plumbing and fixtures has been shown to be ineffective and therefore aiming to completely eradicate potential pathogens from the sink drain environment may not be a suitable approach^23^. A heat and vibration device installed on the p-traps in one study reduced the proportion of carbapenemase-positive sink drain cultures by 80%^24^. Focusing infection control measures on smaller areas, such as the drain cover or tail pipe directly below the drain cover may be just as effective and much less costly.

The *in situ* nature of this study and the focus specifically on sinks allowed us to identify areas of high pathogen concentration and previously unknown differences between sinks in patient rooms and HCP stations. While the culture-dependent methods applied in this study did not capture the microbial community in its entirety, they did allow us to report concentrations of pathogens rather than relative abundance, which is useful for estimating risk of transmission to HCP, patients, or other surfaces. Future work should include culture-independent based analyses of samples similar to those discussed in this study.

## Methods

### Experimental design

Two hospitals located in the same city were subject of this study and henceforth referred to Hospital 1 (a 550-bed acute care facility) and Hospital 2 (an 80-bed acute care facility). Samples from Hospital 1 were collected from a twenty-bed critical care unit and samples from Hospital 2 were collected from a ten-bed critical care unit (Figure S1). Patient rooms at both hospitals were single occupancy only and all sinks had manually operated faucets (no electronic sensor faucets). Hospital standard protocol for both facilities stated that sinks are to be cleaned daily by environmental services using a quaternary ammonium solution (Quat Disinfectant Cleaner Concentrate 5; 3M, St. Paul, MN) applied to microfiber cloths for the sink surface and basin and bleach wipes to clean with sink handles and faucet at Hospital 1; Hospital 2 used a similar quaternary ammonium solution (3M 40 L Disinfectant; 3M, St. Paul, MN) applied to microfiber cloths to clean sink faucet and handles and then sink surface and basin. Sink cleaning protocols were the same for patient room and HCP sinks. Both hospitals received chlorine-treated water from the same municipal source, but Hospital 1 used Ag/Cu as a secondary disinfectant in the hot water supply and Hospital 2 used chlorine dioxide. The sample collection period was divided into two study periods: the intensive sampling period which took place for 6 weeks at each hospital and a long-term sampling period which took place over 11 months at each hospital. During the intensive sampling period, p-trap water was collected Monday through Thursday each week, tap water samples were collected once weekly, and sink surface, drain cover, tail pipe, and p-trap biofilm were collected at 0 weeks, 3 weeks, and 6 weeks. Samples were collected from two patient room sinks and two HCP sink stations at each hospital, with the same four sinks sampled repeatedly at each hospital over the course of the study. For the long-term study, p-trap biofilm samples were collected by removing sink p-traps after 1, 3, 6, 9, and 12 weeks of attachment to the aforementioned sinks, with a new, sterilized p-trap attached at each exchange.

### Sample collection and processing

**Figure 7:**
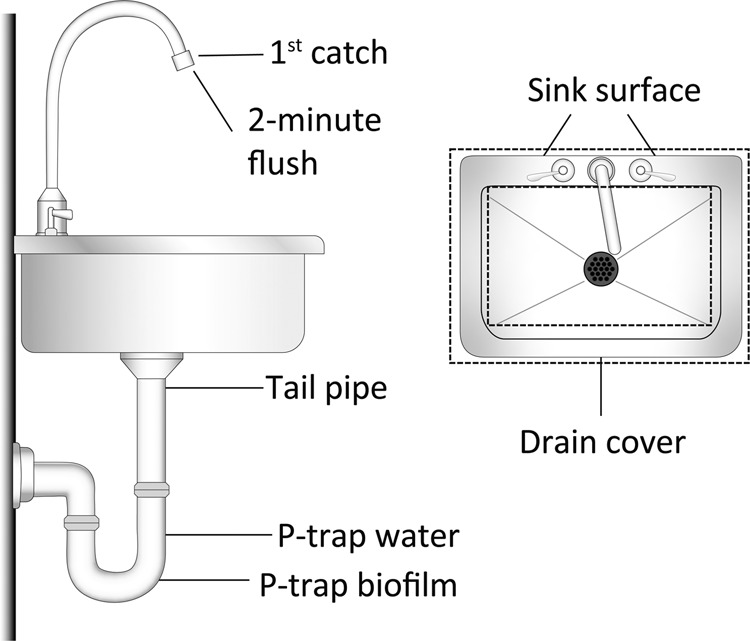
Samples collected from each sink from side and top down views. Sink surface sample included sink handles and area of sink within dashed rectangles.

Samples were transported on ice by overnight courier the day that samples were collected. Samples were processed the following day. The collection points for sample types described below are depicted in Fig. 7. Samples were collected in the following order in order to avoid sample contamination or disruption via the sampling methods: sink surface, drain cover, tail pipe, p-trap water, p-trap, 1^st^ catch, 2-minute flush.

*Tap water*. Tap water samples from the cold water supply were collected in 1 L sterile Nalgene bottles with 1.46% and 1.0% (final concentrations) sodium thiosulfate and sodium thioglycolate, respectively added to water from Hospital 1 to neutralize the copper, silver, and chlorine disinfectants; 20 mg thiosulfate tablets (Safe DChlor T10, Brim Technologies, Eatontown, NJ) were added to water samples from Hospital 2 to neutralize residual chlorine. First-catch samples were defined as the first liter of water to come out of the faucet during the sampling time. At Hospital 1, one patient room sink and one HCP sink had faucet aerators and the one patient room sink and one HCP sink did not. At Hospital 2, all sink faucets had aerators. Two-minute flush samples were defined as one liter of water collected after the tap was flushed with cold water for two minutes. Water temperature, free chlorine, total chlorine, and chlorine dioxide (Hospital 2 only) were measured on-site immediately after collecting tap water samples using Hach Pocket Colorimeter II for free chlorine, total chlorine, and chlorine dioxide with N,N-diethyl-p-phenylenediamine (DPD) reagents, according to the manufacturer’s protocol (Hach, Loveland, CO). Copper and silver was not measured as a part of this study, but Hospital 1 facilities management take monthly samples and adjust the Ag/Cu levels if necessary to fall within the target ranges 0.3–0.8 ppm Cu and 30–80 ppb Ag. Tap water was filtered (0.2 µm pore size), the filter plated on Reasoner’s 2a (R2A) media for heterotrophic plate counts (HPC) and incubated at 25 °C for 7 days. To detect and enumerate rapid growing nontuberculous mycobacteria (NTM), samples were treated with 0.005% cetylpyridinium chloride (CPC) for 30 minutes, filtered, the filter plated on 7H11 Middlebrook agar, and incubated at 30 °C. Plates were counted at 3, 5, and 7 days. Slow growing NTM were not measured.

#### Sink surface and drain cover

Sink surface was defined as the faucet handles and the area on either side and in front of the sink basin that is enclosed in the dashed rectangle in Fig. 1. 3 M^TM^ Sponge-sticks pre-moistened with neutralizing buffer (3M, St. Paul, MN), were used to sample the sink surface by wiping the sponge across the area using multidirectional and overlapping motions. The drain cover was sampled with a separate sponge-stick in the same manner described above. Sponge-sticks were processed according to Rose *et al*.^25^, with the following modifications: 45 ml of phosphate buffered saline with 0.02% Tween 80 (PBST) was added to the stomacher bags and processed in a Stomacher 400 Circulator (Seward, Bohemia, NY) at 230 rpm for 1 minute. Eluent was concentrated by centrifugation at 2700 × g for 20 min.

#### Tail pipe

The section of pipe below the drain cover and above the p-trap was sampled using an ESwab^TM^ (Copan, Murrieta, CA) premoistened in the supplied Amies transport solution buffer. ESwabs^TM^ were inserted through the holes in the drain cover at a depth of 3 inches and the inner wall of the pipe was sampled with four swabs. The swabs were stored in 1 ml Amies transport solution for transport to the laboratory. ESwabs^TM^ were processed by transferring swabs and transport medium to a 50 ml conical tube containing 20 ml PBST. Tubes were vortexed in 10 second bursts for 2 minutes total and then the homogenate was measured and plated on the selective and non-selective media types described below.

#### P-trap water

P-trap water was sampled using a sterile piece of silicone tubing attached to a sterile syringe. The tubing was fed down through the drain cover and then the p-trap water was drawn into the syringe^11^. The water was expelled into a sterile Nalgene bottle and the process was repeated until all water was withdrawn.

#### P-trap

After p-trap water was collected, the p-trap was removed, a sterile cap that was made by fitting a piece of 1/8 inch thick silicone rubber into the p-trap nut, was put on the short end of the p-trap. Butterfield buffer, pH 7.2, was poured into the p-trap to fill the entire volume and another cap was secured to the tall end of the p-trap, creating a water tight seal. To recover the biofilm from the inner walls of the p-trap, the Butterfield buffer and any dislodged biofilm was collected in a sterile bottle during laboratory processing. Two sponges that were removed from Sponge-sticks (one per p-trap opening) and held with sterile forceps were used to wipe the inner surface using a twisting motion. Sponge-sticks were processed as described above, but with 90 ml PBST in stomacher bags instead of 45 ml. Twenty ml PBST was added to the p-trap, caps were re-attached, and p-traps were gently shaken to rinse all inner surfaces. This volume, along with the Sponge-stick eluent were combined with the Butterfield buffer collected from the p-trap. The final volume was measured, centrifuged at 2700 × g for 20 min, and concentrated to approximately 100 ml.

#### Field blank

One unused Sponge-stick per sink per sampling time point was used as a control. The Sponge-stick was removed from the packaging and moved in a manner similar to the sink surface sampling without touching a surface. The Sponge-stick was processed as described above.

#### Culture conditions

All of the samples above, excluding the tap water, were plated on R2A, Middlebrook 7H11 (Remel, Lenexa, KS), Pseudosel (Becton Dickinson, Franklin Lakes, NJ), Chromagar KPC (Chromagar, Paris, France), and MacConkey with 2 mg/L cefotaxime (Hardy Diagnostics, Santa Maria, CA). R2A plates were incubated at 25 °C for 7 days; 7H11 Middlebrook plates were incubated at 30 °C and counted at 3, 5, and 7 days (all samples were CPC treated as described above prior to plating on 7H11 Middlebrook medium); Pseudosel plates were incubated at 35 °C for 24 hrs and then at 25 °C for an additional 24 hrs; Chromagar KPC plates were incubated for 24 hours at 35 °C; and MacConkey with 2 mg/L cefotaxime plates were incubated at 35 °C for 72 hrs. Volume plated was maximum 5 ml (concentrated on a filter), making the limit of detection 6 CFU/ml given a countable plate definition of 30–300 colonies. Final bacterial counts factored in dilutions and the original volume collected or surface area sampled.

#### Plate count confirmation and isolate identification

For Pseudosel (targeting *P. aeruginosa*) and Chromagar KPC (targeting CRE) media, colonies suspected to be target organisms were isolated and identified using matrix-assisted laser desorption ionization time-of-flight (MALDI-ToF) mass spectrometry (Bruker Daltonik MALDI biotyper; Bruker, Bremen, Germany)^26^. One of every colony morphology that grew on the MacConkey with cefotaxime agar (lactose fermenting and non-lactose fermenting) were isolated and identified by MALDI-ToF. Plate counts on MacConkey with cefotaxime agar represent *Pseudomonas* spp (including *P. aeruginosa)*, Enterobacteriaceae, *Achromobacter* spp., *Stenotrophomonas maltophilia*, *Acinetobacter* spp., and other organisms that comprised <1.5% of isolates recovered from this medium. Isolates from this medium correspond well with the list of organisms in CDC Division of Healthcare Quality Promotion summary of CDC consultations involving healthcare infection risks from water-related organisms^1^.

### Antibiotic susceptibility testing

Isolates that were identified as *P. aeruginosa* or members of the Enterobacteriaceae family were selected for antibiotic susceptibility testing (AST) via disk diffusion according to Clinical and Laboratory Standards Institute (CLSI) guidelines^27^. *P. aeruginosa* isolates were tested against Aztreonam, Ceftazidime, and Meropenem; Enterobacteriaceae were tested against Aztreonam, Ceftazidime, Meropenem, Ertapenem, and Cefotaxime. Isolates that were determined to exhibit resistance or intermediate resistance (as per the most recent CLSI breakpoints) to any of these antimicrobial agents were designated as ARO for the purposes of this study.

### Statistical analyses

Plate count data was compared between HCP and patient room sinks by using the Wilcoxon signed-rank test due to the non-normal distribution of the data. Plate count data from patient and HCP sinks were paired so that data from a patient sink was always compared to the same HCP sink that was sampled on the same day (when all sample types were collected from a sink, two sinks were sampled per day in two consecutive days). AST data was compared using 3-way ANOVAs with antibiotic, sample type (biofilm vs. planktonic), and sink type (patient vs HCP) as the factors. AST data was determined to be normal by the Shapiro-Wilk test.

## Supplementary information

Supplemental Figures.

## Data Availability

The datasets generated during and/or analyzed during the current study are available from the corresponding author on reasonable request.

## References

[1] Perkins KM, Reddy SC, Fagan R, Arduino MJ, Perz JF (2019). Investigation of healthcare infection risks from water-related organisms: Summary of CDC consultations, 2014-2017. Infect Control Hosp Epidemiol.

[2] Williams MM, Armbruster CR, Arduino MJ (2013). Plumbing of hospital premises is a reservoir for opportunistically pathogenic microorganisms: a review. Biofouling.

[3] Kanamori H, Rutala WA, Gergen MF, Weber DJ (2016). Patient Room Decontamination against Carbapenem-Resistant Enterobacteriaceae and Methicillin-Resistant Staphylococcus aureus Using a Fixed Cycle-Time Ultraviolet-C Device and Two Different Radiation Designs. Infect Control Hosp Epidemiol.

[4] Weingarten, R. A. *et al*. Genomic Analysis of Hospital Plumbing Reveals Diverse Reservoir of Bacterial Plasmids Conferring Carbapenem Resistance. *MBio* **9**, 10.1128/mBio.02011-17 (2018). 10.1128/mBio.02011-17 PMC5801463 29437920

[5] Tofteland S, Naseer U, Lislevand JH, Sundsfjord A, Samuelsen O (2013). A long-term low-frequency hospital outbreak of KPC-producing Klebsiella pneumoniae involving Intergenus plasmid diffusion and a persisting environmental reservoir. PLoS One.

[6] Roux D (2013). Contaminated sinks in intensive care units: an underestimated source of extended-spectrum beta-lactamase-producing Enterobacteriaceae in the patient environment. J Hosp Infect.

[7] Leitner E (2015). Contaminated handwashing sinks as the source of a clonal outbreak of KPC-2-producing Klebsiella oxytoca on a hematology ward. Antimicrob Agents Chemother.

[8] Lowe C (2012). Outbreak of extended-spectrum beta-lactamase-producing Klebsiella oxytoca infections associated with contaminated handwashing sinks(1). Emerg Infect Dis.

[9] Breathnach AS, Cubbon MD, Karunaharan RN, Pope CF, Planche TD (2012). Multidrug-resistant Pseudomonas aeruginosa outbreaks in two hospitals: association with contaminated hospital waste-water systems. J Hosp Infect.

[10] Grabowski M (2018). Characterizations of handwashing sink activities in a single hospital medical intensive care unit. J Hosp Infect.

[11] Kotay, S. M. *et al*. Droplet- Rather than Aerosol-Mediated Dispersion Is the Primary Mechanism of Bacterial Transmission from Contaminated Hand-Washing Sink Traps. *Appl Environ Microbiol* **85**, 10.1128/AEM.01997-18 (2019). 10.1128/AEM.01997-18 PMC6328769 30367005

[12] Kotay, S., Chai, W., Guilford, W., Barry, K. & Mathers, A. J. Spread from the Sink to the Patient: *In Situ* Study Using Green Fluorescent Protein (GFP)-Expressing Escherichia coli To Model Bacterial Dispersion from Hand-Washing Sink-Trap Reservoirs. *Appl Environ Microbiol* **83**, 10.1128/AEM.03327-16 (2017). 10.1128/AEM.03327-16 PMC5377511 28235877

[13] Lenhart TR (2014). Identification and characterization of microbial biofilm communities associated with corroded oil pipeline surfaces. Biofouling.

[14] Martiny AC, Jorgensen TM, Albrechtsen HJ, Arvin E, Molin S (2003). Long-term succession of structure and diversity of a biofilm formed in a model drinking water distribution system. Appl Environ Microbiol.

[15] Gomez-Alvarez V, Revetta RP, Santo Domingo JW (2012). Metagenome analyses of corroded concrete wastewater pipe biofilms reveal a complex microbial system. BMC Microbiol.

[16] Lehtola MJ (2006). The effects of changing water flow velocity on the formation of biofilms and water quality in pilot distribution system consisting of copper or polyethylene pipes. Water Res.

[17] Hennequin C, Aumeran C, Robin F, Traore O, Forestier C (2012). Antibiotic resistance and plasmid transfer capacity in biofilm formed with a CTX-M-15-producing Klebsiella pneumoniae isolate. J Antimicrob Chemother.

[18] Savage VJ, Chopra I, O’Neill AJ (2013). Staphylococcus aureus biofilms promote horizontal transfer of antibiotic resistance. Antimicrob Agents Chemother.

[19] United States Environmental Protection Agency, *National Primary Drinking Water Regulations*. 2009.

[20] Denton M, Mooney L, Kerr KG (2000). Faucet aerators: a source of patient colonization with Stenotrophomonas maltophilia. Am J Infect Control.

[21] Cristina ML (2014). The impact of aerators on water contamination by emerging gram-negative opportunists in at-risk hospital departments. Infect Control Hosp Epidemiol.

[22] Lax, S. *et al*. Bacterial colonization and succession in a newly opened hospital. *Sci Transl Med* **9**, 10.1126/scitranslmed.aah6500 (2017). 10.1126/scitranslmed.aah6500 PMC5706123 28539477

[23] Gbaguidi-Haore H (2018). A Bundle of Measures to Control an Outbreak of Pseudomonas aeruginosa Associated With P-Trap Contamination. Infect Control Hosp Epidemiol.

[24] Mathers AJ (2018). Intensive Care Unit Wastewater Interventions to Prevent Transmission of Multispecies Klebsiella pneumoniae Carbapenemase-Producing Organisms. Clin Infect Dis.

[25] Rose LJ, Hodges L, O’Connell H, Noble-Wang J (2011). National validation study of a cellulose sponge wipe-processing method for use after sampling Bacillus anthracis spores from surfaces. Appl Environ Microbiol.

[26] Clark AE, Kaleta EJ, Arora A, Wolk DM (2013). Matrix-assisted laser desorption ionization-time of flight mass spectrometry: a fundamental shift in the routine practice of clinical microbiology. Clin Microbiol Rev.

[27] Clinical and Laboratory Standards Institute (CLSI). *Performance Standards for Antimicrobial Susceptibility Testing*. 28th ed. CLSI supplement M100. Clinical and Laboratory Standards Institute, 950 West Valley Road, Suite 2500, Wayne, Pennsylvania 19087 USA, 2018.

