# Supplementary material for: A microbiological survey of handwashing sinks in the hospital built environment reveals differences in patient room and healthcare personnel sinks
Source: Sci Rep. 2020 May 19;10:8234. doi: 10.1038/s41598-020-65052-7 (PMC7237474; doi:10.1038/s41598-020-65052-7)
Supplement: Supplementary file 1 — Supplemental Figures. [file 41598_2020_65052_MOESM1_ESM.pdf]

Supplemental Information for:

A microbiological survey of handwashing sinks in the hospital built environment reveals differences in patient room and healthcare personnel sinks

Authors: Lauren C. Franco[1,3], Windy Tanner[2], Christine Ganim[1], Terri Davy[1], Jonathan Edwards[1], Rodney Donlan[1]

Affiliations: [1]Centers for Disease Control and Prevention, Atlanta, GA USA
[2]University of Utah, Salt Lake City, UT USA
[3]Oak Ridge Institute for Science and Education, Oak Ridge, TN USA

Organism	# of Isolates
Pseudomonas spp. (other than aeruginosa)	294
Pseudomonas aeruginosa	195
Stenotrophomonas maltophilia	111
Sphingobacterium spp.	53
Enterobacter spp.	42
Delftia spp.	26
Achromobacter spp.	19
Brevundiomonas spp.	18
Ochrobactrum spp.	8
Acinetobacter spp.	7
Chryseobacter spp.	7
Elizabethkingia spp.	7
Rhizobium spp.	7
Citrobacter spp.	6
Cupriavidus spp.	6
Klebsiella spp.	4
Pannonibacter phragmitetus	1
Serratia marcesens	1

Table S1. List of isolate identities from Chromagar KPC, Pseudosel, and MacConkey + 2mg/L CTX selective media. Number of isolates belonging to each genus is listed and is total number of isolates across all samples collected.

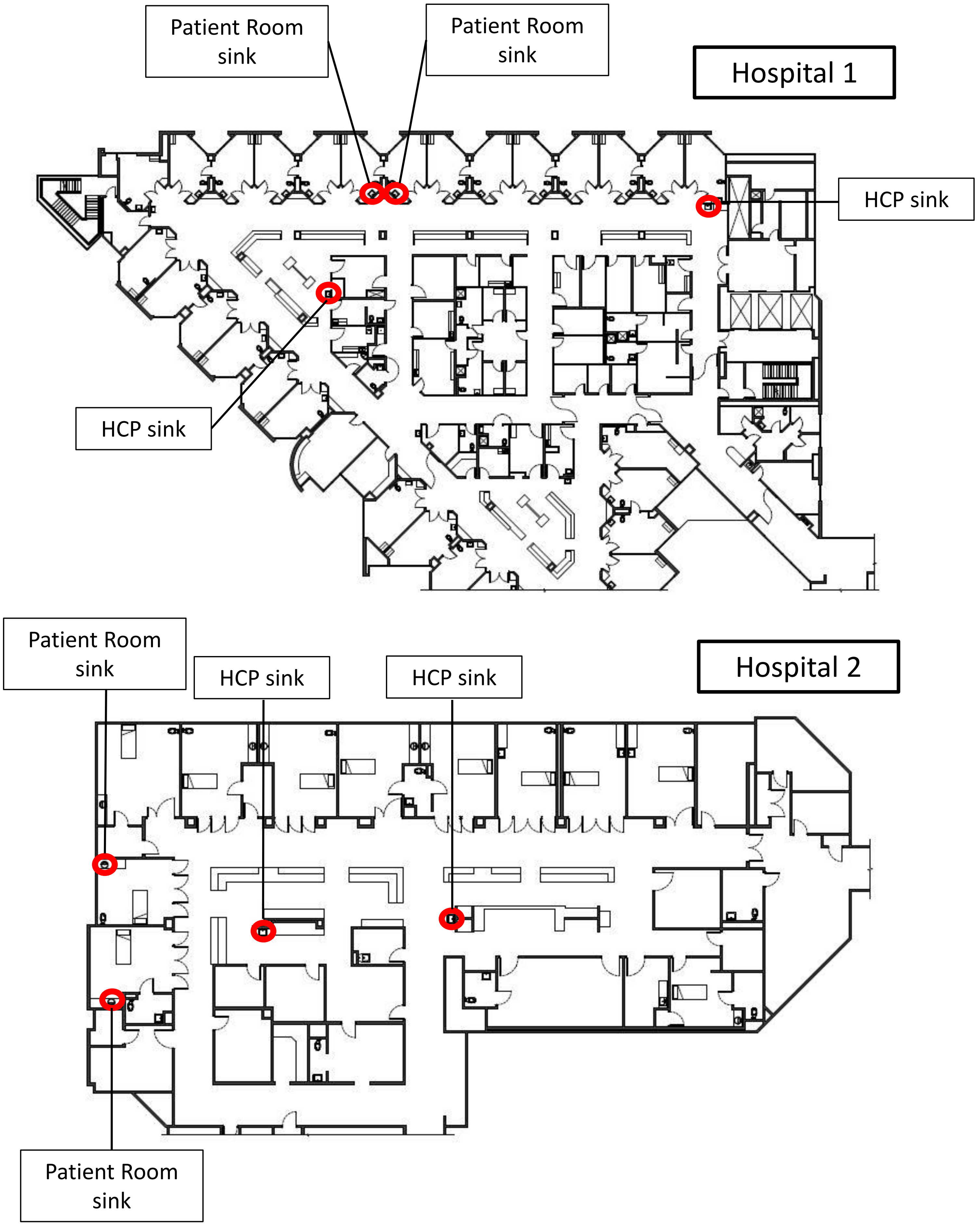

Supplemental Figure 1. Floorplans highlighting locations of patient room and healthcare personnel (HCP) sinks that were sampled in Hospital 1 and Hospital 2.
